# Ribonucleic Acid Engineering of Dendritic Cells for Therapeutic Vaccination: Ready ‘N Able to Improve Clinical Outcome?

**DOI:** 10.3390/cancers12020299

**Published:** 2020-01-27

**Authors:** Yannick Willemen, Maarten Versteven, Marc Peeters, Zwi N. Berneman, Evelien L. J. Smits

**Affiliations:** 1 Laboratory of Experimental Hematology, VAXINFECTIO, Faculty of Medicine and Health Sciences, University of Antwerp, 2610 Antwerp, Belgium; 2 Center for Oncological Research, Faculty of Medicine and Health Sciences, University of Antwerp, 2610 Antwerp, Belgium

**Keywords:** dendritic cell, vaccination, immunotherapy, ribonucleic acid, cancer, human immunodeficiency virus

## Abstract

Targeting and exploiting the immune system has become a valid alternative to conventional options for treating cancer and infectious disease. Dendritic cells (DCs) take a central place given their role as key orchestrators of immunity. Therapeutic vaccination with autologous DCs aims to stimulate the patient’s own immune system to specifically target his/her disease and has proven to be an effective form of immunotherapy with very little toxicity. A great amount of research in this field has concentrated on engineering these DCs through ribonucleic acid (RNA) to improve vaccine efficacy and thereby the historically low response rates. We reviewed in depth the 52 clinical trials that have been published on RNA-engineered DC vaccination, spanning from 2001 to date and reporting on 696 different vaccinated patients. While ambiguity prevents reliable quantification of effects, these trials do provide evidence that RNA-modified DC vaccination can induce objective clinical responses and survival benefit in cancer patients through stimulation of anti-cancer immunity, without significant toxicity. Succinct background knowledge of RNA engineering strategies and concise conclusions from available clinical and recent preclinical evidence will help guide future research in the larger domain of DC immunotherapy.

## 1. Introduction

### 1.1. Dendritic Cell (DC) Vaccination

Dendritic cells (DCs) are key regulators of the immune system which precisely and specifically direct the immune response against antigens [1,2]. Given their central role in immunity, they are being actively investigated worldwide in preclinical and clinical studies as a vaccine strategy [1,2]. DC vaccination is a form of active specific immunotherapy, aiming to stimulate the host’s immune system in vivo to develop a strong and lasting immune response against disease-associated antigens (DAAs). Subject to human leukocyte antigen (HLA) restrictions, DC vaccines are generally tailored to each individual patient in an autologous context using monocyte-derived (mo)-DCs. A long story short, this renders DC vaccination the status of a complex and laborious approach yet with a high degree of precision resulting in virtually no significant side effects.

### 1.2. Disease Applications

DC vaccination may evidently target microbial pathogens, but also autologous threats such as tumor cells. With less complicated vaccine strategies already proving highly effective against numerous infectious diseases, DC vaccination has especially found a firm foothold in cancer over the past 20 years. The observation that the immune system can recognize and eliminate malignant cells has fueled a truly exponential growth in tumor immunology research and the clinical study of cancer immunotherapy, including DC vaccination. Since the first trial in 1996, DC vaccines have been repeatedly found capable of inducing clinical responses in cancer patients [2]. While sparking hope in terms of increased overall survival, objective responses are limited to a disappointingly small minority and investigations have been ongoing to improve outcomes [2].

### 1.3. Optimizing Efficacy

The ideal DC-based cancer vaccine contains highly immunogenic DCs that can activate a broad repertoire of tumor-specific T cells through presentation of multiple major histocompatibility complex (MHC) class I and II-restricted epitopes of different DAAs. In addition, however, it has become increasingly evident that the clinical success of DC-based cancer vaccination—and cancer immunotherapy in general—is hampered by cancer cell immune escape and immune inhibition [3,4]. Therefore, besides loading DCs with an appropriate DAA, attention has been directed toward boosting DC’s immune-stimulatory properties as well as rendering them capable of withstanding or circumventing immune-inhibitory signaling.

### 1.4. Ribonucleic Acid (RNA) Modification

A continuously popular approach to potentiate vaccine DCs involves modifying the DCs’ gene expression profile by introducing exogenous RNA. As will be discussed further, RNA offers certain advantages which make it a very versatile, safe, and effective method to engineer DCs. Several clinical studies have explored the clinical efficacy of different RNA-modified DC vaccines in various disease settings. The results of these studies and lessons learned from them will be discussed in this review, together with an outlook on ongoing and developing strategies to improve DC vaccine efficacy.

## 2. RNA Sources and Products

From a conceptual viewpoint, using RNA encoding the full-length antigen—as compared to peptides—to load vaccine DCs with DAAs requires no prior epitope definition, making it broadly applicable across all HLA genotypes and potentially allowing both MHC class I and II presentation depending on intracellular targeting. In the early days of modifying DCs through RNA, nearly two decades ago, total RNA or messenger (m)RNA isolated from tumor cells was most often used to load DCs with tumor-associated antigens (TAAs). Developing technology and theoretical concerns about expression of other tumor-related and perhaps oncogenic genes drove a shift towards the use of defined synthetic RNAs. They offer the advantage of knowing beforehand exactly what is being inserted into the cell, but also expand the possibilities. Introducing DAAs—nowadays usually through synthetic mRNA—remains by far the most applied strategy for RNA-based engineering of DCs used in clinical vaccination, as shown further. Yet, mRNAs encoding other genes and interfering RNAs such as small interfering (si)RNA, short hairpin (sh)RNA, or micro (mi)RNA are increasingly gaining attention both in preclinical and clinical research as ways to manipulate the DC’s gene expression profile for optimizing vaccine efficacy.

### 2.1. Total RNA/mRNA

The use of total RNA or mRNA is largely restricted to the setting of cancer. Twenty years ago, when fewer tumor antigens had been identified and in vitro RNA synthesis still had to take off, this approach offered a way of loading DCs with TAAs. Moreover, head-to-head comparisons with tumor-cell (lysate) pulsing or DC hybrid fusion showed that the use of total (m)RNA to load DCs increased T-cell numbers and function, autologous tumor-cell lysis in vitro in cancer patients, and tumor growth delay in vivo in mice [5,6,7,8,9,10,11]. In the current age, loading DCs with total RNA/mRNA is still often used, for the same reasons as in 1998: it could offer a broader antitumor immune response and counter the emergence of antigen-loss variants and natural immune variation [12]. A drawback of this approach is that it requires sufficient autologous tumor tissue, which poses a constraint even despite techniques such as (m)RNA amplification from single cells.

### 2.2. Synthetic RNA

In vitro transcribed (IVT) or generated synthetic RNAs are used for regulating the protein expression of vaccine DCs to fine-tune immune responses. mRNA serves as a template for target antigens and desirable immune-stimulatory proteins such as cytokines and co-stimulatory molecules. Conversely, synthetic interfering RNA, in the form of siRNA, shRNA, or miRNA, is designed to downregulate the expression of unwanted immune-inhibitory signals.

### 2.3. Messenger RNA (mRNA)

To date, mRNA transfection is by far the most popular strategy for modifying vaccine DCs used in clinical trials. Its technical success is owed to its simplicity, safety, and expression efficiency [13]. Protein expression is subject to mRNA stability, localization in the cell, and translation [14]. By optimizing structural mRNA characteristics such as the 5’ cap, the poly(A) tail, and the codon sequence, combined with highly efficient transfection methods (e.g., electroporation or lipofection), protein expression can be greatly increased [15]. Expression is always transient, without disruption of the host’s genome. While advantageous for safety reasons, this may also be a disadvantage, though this only seems relevant when DCs are transfected several days before vaccination (e.g., in their immature state), because expression generally lasts up to two to five days [16].

Table 1 lists all mRNA products that have been examined in RNA-modified DC vaccination clinical trials and how many subjects have been vaccinated with each. Besides a wide array of TAAs, including a large share of melanoma-associated antigens, the list also contains viral antigens, primarily from human immunodeficiency virus (HIV)-1 and for a lesser amount from cytomegalovirus (CMV). As shown in detail in the Table S1, in several clinical trials the antigen-encoding mRNA was fused to lysosome-associated membrane protein (LAMP)-1 mRNA to direct antigen presentation towards the MHC class-II signaling pathway to enhance stimulation of CD4+ T-cell responses [17,18]. Finally, there have been clinical trials using co-electroporation of immature mo-DCs with co-stimulatory molecules CD40L and CD70 mRNA and constitutively-active Toll-like receptor-4 (cTLR4) mRNA as danger signal to generate mature DCs (so-called TriMix DCs), which are capable of inducing strong antigen-specific T-cell responses in vitro and in vivo after co-electroporation together with TAA-encoding mRNA [19].

In a preclinical setting, numerous other antigen mRNA-loaded DCs have been investigated in in vitro human and in vivo animal studies. There is a seemingly exhaustive list of TAAs, including microbial antigens (e.g., human papillomavirus, hepatitis C virus, and fungal antigens), as well as self-antigens expressed on regulatory T cells (e.g., FoxP3) or involved in tumor growth (e.g., fibroblast stroma and angiogenic factors) [17,20,21,22,23,24,25,26]. Such extensive worldwide research efforts are driven by the most important feature of DAA-encoding mRNA transfection, which is that it can result in the presentation of different (undefined) epitopes via both MHC class I and II pathways, activating different CD4+ and CD8+ T-cell clones responsible for effective anti-disease immunity in vivo.

Next to antigen-encoding mRNA, studies on RNA modification of DCs to enhance vaccine efficiency explore the effects of mRNA encoding immune-regulatory cytokines or co-stimulatory molecules. A number of preclinical studies with murine tumor models reported in vivo antitumor effects of immunization with DCs genetically modified to express a variety of cytokines, including granulocyte-macrophage colony-stimulating factor (GM-CSF), interleukin (IL)-2, IL-7, IL-12, IL-21, IL-23, IL-32, and interferon (IFN)-α [27,28,29,30,31,32,33,34,35,36]. All these studies relied on viral transduction to modify the DCs’ cytokine secretion profile, largely explaining why only one clinical trial has been conducted so far with DCs that were virally transduced to produce IL-12, despite such a broad preclinical evidence base [37]. In vitro human studies demonstrated that co-electroporation of DCs with IL-12, IL-15 + IL-15Rα or IFN-α mRNA, in addition to TAA-encoding mRNA, boosts antitumoral NK-cell and T-cell activation and effector functions [38,39,40,41]. Additionally, the introduction of mRNA encoding co-stimulatory molecules CD40L, CD137L, or CD134L (CD252/OX40L) into DCs was shown to increase CD4+ and CD8+ T-cell responses in vitro and antitumor immunity in vivo [42,43]. Another strategy aimed at improving lymph node targeting of DCs following vaccination by loading them with mRNA encoding a human chimeric E/L-selectin (CD62E/CD62L) protein which binds to peripheral node addressin in endothelial venules. These E/L-selectin mRNA-electroporated DCs gained the ability to attach and roll on sialyl-Lewis(X) in vitro while maintaining their phenotype and regular CCR7-mediated migratory and TAA-specific cytotoxic T lymphocyte-stimulating capacities [44].

In summary, mRNA products under investigation for modifying vaccine DCs are for the largest part TAAs, but also other DAAs such as microbial antigens, or immune-regulatory proteins such as cytokines and co-stimulatory molecules. Exogenously introduced mRNA can also code for proteins that activate and mature DCs. Notably, there might be a limit to what can be force-fed to the cell’s translational machinery, considering a report of individual transgene protein expression slightly decreasing after simultaneous transfection with three different mRNAs [45].

### 2.4. Interfering RNA (shRNA/siRNA)

Different genes have already been targeted by interfering RNAs to reduce their expression in DCs: pro-apoptotic factors BAK, BAX, BIM, CED-3, CED-4, immune-inhibitory signals programmed death ligand (PD-L)1/2, IL-10, scavenger receptor A (SRA/CD204), indoleamine 2,3-deoxygenase (IDO) and suppressor of cytokine signaling (SOCS)-1, and the inducible immunoproteasome (iP).

Downregulating the expression of pro-apoptotic factors such as BAK/BAX, BIM, CED-3, or CED-4 in DCs through siRNA improved DC survival and enhanced their therapeutic efficacy in mouse tumor models [46,47,48]. Silencing inhibitory checkpoint molecules PD-L1 and PD-L2 in mo-DCs through siRNA could be combined with TAA peptide pulsing or mRNA electroporation and increased antigen-specific CD8+ T cells expansion from cancer patients ex vivo and mice in vivo [49,50]. Similarly, loading DCs with shRNA against the ubiquitin-editing protein A20, which is an important downregulator of inflammatory signaling molecule nuclear factor-κB (NF-κB), could be combined with antigen mRNA loading and resulted in greater antitumor activity in vivo in a melanoma mouse model [51]. Targeting expression of other immunosuppressive factors IL-10, SRA/CD204, IDO, and SOCS-1 in DCs through siRNA also boosted antigen-specific T-cell responses in vivo in rodents and/or patients [52,53,54,55]. Furthermore, intratumoral vaccination with SRA/CD204-silenced DCs, compared to unmodified DCs, synergized with radiotherapy to reduce tumor growth and lengthen survival in a prostate cancer mouse model [56]. In contrast, siRNA knockdown of DC-SCRIPT in DCs modified cytokine production following TLR stimulation with more IL-10 and less IL-12 secretion [57]. Despite these many promising in vitro and preclinical animal in vivo results, only siRNA targeting three inducible iP subunits or IDO has been used in clinical trials with RNA-modified DC vaccination so far (Table 1 and Table S1).

### 2.5. MicroRNA (miRNA)

There is a great hype in research these days about the role of microRNAs in various pathologies. While this may eventually lead to potential therapeutic targets to modulate DC function, there is currently no actual application of miRNAs to engineer DCs [58].

## 3. RNA Delivery Methods

Several strategies have been effectively employed to load DCs with RNA, with the simplest one of passive pulsing starting two decades ago and new techniques still being developed. Already in 1996, Boczkowski et al. reported resistance to tumor challenge and reduced tumor growth in ovalbumin-expressing tumor and melanoma mouse models after vaccination with bone marrow-derived (BM)-DCs that were passively pulsed with IVT ovalbumin mRNA and naked tumor mRNA [59].

Due to low transfection efficiency other, active ways of introducing RNA into DCs were explored from around the same time in the late 1990s. Viral transduction of DCs, in which a virus is modified to contain a gene of interest and then used to infect DCs and insert that gene, is a much more efficient gene transfer method with high transgene expression [60,61]. This is strictly not an RNA transfer method, but is worth mentioning briefly as it has been and still is often applied to genetically engineer DCs, though almost exclusively in vitro or in preclinical in vivo animal studies. Compared to other methods, viral transduction can be very laborious, and its clinical application has been hindered by (theoretical) disadvantages such as potential host genome disruption and anti-viral host immune responses neutralizing the vaccine or competing with desirable immunity against DAAs [61]. Walch et al. described a recombinant yeast transduction method which they found to be safer and more efficacious in vitro to deliver functional mRNA into human mo-DCs compared to many bacterial or viral techniques [62,63]. However, any method using microbial vectors still harbors the same (theoretical) concerns for clinical application as does viral transduction.

In 2001, our own group showed that mRNA electroporation was a highly efficient method for TAA-loading of mo-DCs, significantly more so than mRNA lipofection, passive pulsing, or cDNA electroporation [16]. This technique revolves around generating an electric field around the DCs in suspension by a short electric pulse of only a few milliseconds, creating transient pores in the cell membrane which allow diffusion of RNA molecules into the cytoplasm. Side-by-side comparisons to viral transduction further proved electroporation to be an equal alternative in terms of mRNA transfection/transduction efficiency and expression [64,65]. The high efficiency, combined with its simplicity in use, favorable safety profile, and relative ease of incorporation into good manufacturing and clinical practice protocols, have put mRNA electroporation on the absolute forefront of RNA-modification of DCs for clinical application.

Other non-viral (or non-microbial) methods for loading DCs with RNA have been examined, with some being primed further for clinical application. Of these, chemical transfection through lipofection is the most evolved and widely used alternative to electroporation, which is now even finding its way into DC vaccination clinical trial protocols for PD-L1/2 siRNA transfection (https://clinicaltrials.gov/ct2/show/NCT02528682). Lipofection or liposome transfection uses phospholipid bilayer vesicles that carry RNA and are then fused with the DC’s cell membrane to insert its contents into the cytoplasm. Next to lipofection, chemical transfection of DCs has also been effectively tried using cationic, hydrophilic, and pH-responsive endosomolytic triblock copolymer segments, as well as non-lipid cationic reagents [21,66]. Transfection efficiency of chemical transfection methods is lower compared to electroporation, but in some studies this was associated with higher transgene protein expression per cell, which in turn yielded stronger immune responses in vitro and in a recombinant vaccinia virus mouse model [21,67]. Interestingly, our own group demonstrated that siRNA lipofection and mRNA electroporation can be combined to engineer classical mo-DCs for cancer vaccination purposes while maintaining their characteristic phenotype [50].

In more recent years, exciting novel techniques have emerged which should ultimately allow for RNA transfection of DCs in vivo. Sonoporation employs microbubbles, consisting of a gas core surrounded by a lipid or polymer shell and loaded with complexes of RNA and cationic liposomes, which eventually implode under ultrasound, resulting in short-lived pores in nearby cells’ membranes and allowing delivery of mRNA to their cytoplasm. Sonoporation of DCs with mRNA-lipoplex microbubbles displayed low transfection efficiency (11%–24%), but also maintained good DC viability [68,69]. Immunization with DCs sonoporated with TAA mRNA and immune-stimulatory TriMix mRNA reduced tumor growth and increased survival in ovalbumin-expressing tumor mouse models [69]. Interestingly, ultrasound-contrast microbubbles were found to migrate to tumor-draining lymph nodes following intradermal injection around breast cancer lesions, thus offering potential for in vivo RNA sonoporation of DCs [70]. Additionally, cell-penetrating peptides (CPPs) are being examined for their potential of targeting and modulating DCs in vivo [71]. CPPs are short amino acids that can carry other amino or nucleic acids into target cells mainly by endocytosis. By design these CPPs should then escape from the endosome before lysosome fusion and diffuse into the cytoplasm. While CPPs have only been used to load DCs with antigen peptides so far, they could also be used to transfect DCs with siRNA as they can other cells [72].

## 4. Clinical Trials

From 2001, there are a total of 52 Medline-indexed publications on clinical trials with RNA-modified DC vaccination detailing results on 835 study subjects (Table S1). Corrected for placebo groups and the considerable overlap in these reports, mainly because of lengthened follow-up periods, they contain data of 696 different subjects vaccinated with RNA-modified DCs. All trials were pilot or phase I and/or II studies, of which only six had a randomized controlled trial (RCT) design; there are no phase III studies so far.

### 4.1. Diseases

**Figure 1:**
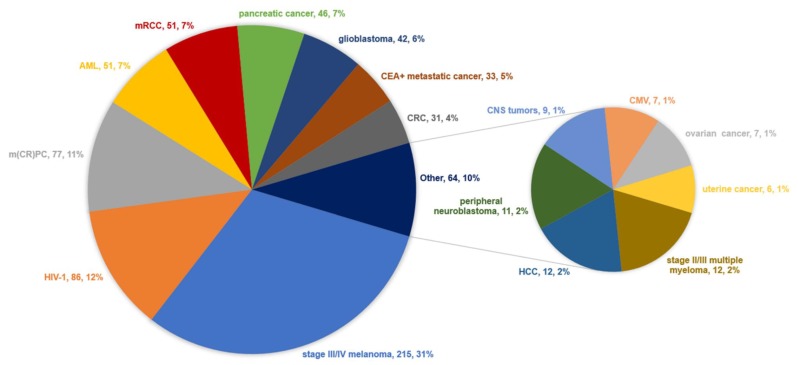
Distribution of all patients vaccinated in clinical trials with RNA-modified dendritic cells per disease setting, including the number and percentage of total (*n* = 696). Abbreviations: AML, acute myeloid leukemia; CEA, carcinoembryonic antigen; CMV, cytomegalovirus; CNS, central nervous system; CRC, colorectal cancer; HCC, hepatocellular carcinoma; HIV-1, human immunodeficiency virus-1; m(CR)PC, metastatic (castration-resistant) prostate cancer; mRCC, metastatic renal cell carcinoma; RNA, ribonucleic acid.

The vast majority of clinical trials examined RNA-modified DC vaccination in the context of cancer, collecting data on more than 600 vaccinated cancer patients (*n* = 602; 87%; Figure 1). Over a third of these patients suffered from stage III or IV melanoma (*n* = 215; 31%). Despite so many vaccinated cancer patients, an infectious disease is the second most frequently studied condition with over 80 vaccinated HIV-1 patients (*n* = 86; 12%). Together with metastatic prostate cancer (*n* = 77; 11%), acute myeloid leukemia (AML; *n* = 51; 7%), metastatic renal cell cancer (*n* = 51; 7%), pancreatic cancer (*n* = 46; 7%), glioblastoma multiforme (*n* = 42; 6%), CEA+ metastatic cancer (*n* = 33; 5%), and colorectal cancer (*n* = 31; 4%) these diseases make up 91% of all vaccinated patients. Other, smaller populations include stage II/III multiple myeloma (*n* = 12; 2%), hepatocellular carcinoma (*n* = 12; 2%), adrenal or retroperitoneal neuroblastoma (*n* = 11; 2%), other central nervous system tumors (*n* = 9; 1%), gynecological malignancies as ovarian (*n* = 7; 1%) or uterine cancer (*n* = 6; 1%), and finally CMV (*n* = 7; 1%) as the only other infectious disease besides HIV-1.

### 4.2. Vaccination

**Figure 2:**
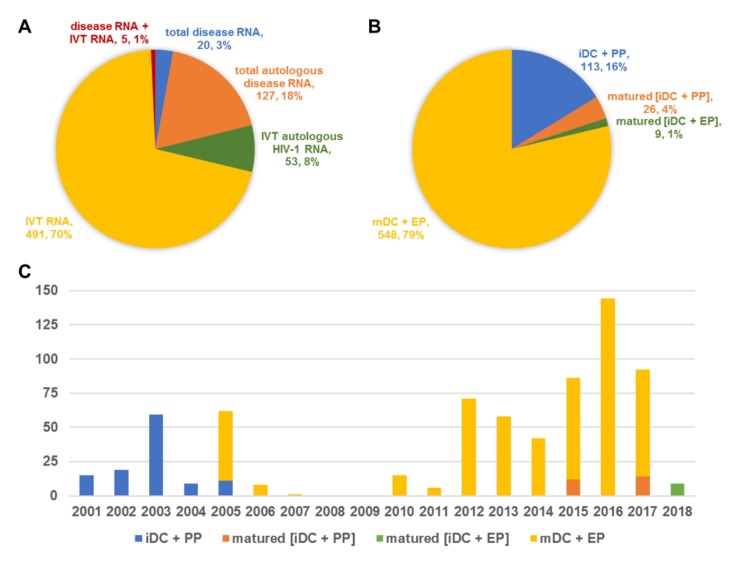
Distribution of all patients vaccinated in clinical trials with RNA-modified dendritic cells per (**A**) RNA source; (**B**) dendritic cell type and RNA loading strategy, including the number and percentage of total (*n* = 696), and (**C**) the spreading of the dendritic cell type and RNA loading strategy over time. Abbreviations: EP, electroporation; HIV-1, human immunodeficiency virus-1; iDC, immature dendritic cells; IVT, in vitro transcribed; mDC, mature dendritic cells; PP, passive pulsing; RNA, ribonucleic acid.

All clinical trials employed in vitro generated autologous mo-DCs for vaccination, differentiated into immature DCs from adherent peripheral blood mononuclear cell (PBMC) fractions or CD14-selected monocytes using GM-CSF and IL-4. In studies using mature mo-DCs, different cytokine cocktails, most often including some combination of tumor necrosis factor-α, IL-1β, IL-6, prostaglandin E2, IFN-γ, or TLR ligands, or even mRNA electroporation itself (i.e., TriMix DCs) were used to ensure DC maturation. Until 2005, published trials (*n* = 8) had used immature DCs that were loaded with RNA by passive pulsing to vaccinate patients (*n* = 113; Figure 2). However, in 2005 there was an impressive shift with reports from then onward (*n* = 43) only describing vaccination of subjects (*n* = 669, of which 583 were unique subjects) using mature DCs. These DCs were either loaded with RNA by passive pulsing in the immature state before maturation (*n* = 35 subjects; 5%) or in most cases through electroporation in the mature stage (*n* = 548 subjects; 79%; Figure 2). The source of the RNA used to load DCs also evolved since 2001, with the use of total (autologous) disease RNA quickly fading in favor of synthetic amplified specific autologous disease RNA or IVT mRNA (Figure 2). All trials used disease-associated (m)RNA, but only two also investigated the effects of siRNA. Finally, vaccination itself was performed with 10^5^ to 10^8^ but mostly around 5–20 × 10^6^ DCs via the subcutaneous, intradermal, intranodal, or intravenous route, often including several initial rounds of vaccination and sometimes followed by so-called booster vaccinations at later times with larger intervals (Table S1).

The pharmacodynamic effects of RNA-modified DC vaccines, in this case the immunological effects, and the clinical effects and safety/toxicity observed in the various clinical trials are described in the corresponding sections below. Regarding their pharmacokinetic properties, there are no conventional absorption, distribution, metabolism, and excretion (ADME) studies. Clinical trials employed specific vaccine release criteria based on viability and phenotype (not detailed here). In 2003, De Vries et al. studied in vivo trafficking of peptide-loaded immature versus mature DCs towards distant lymph nodes after intradermal versus intranodal injection in melanoma patients [73]. They found that mature DCs migrated more to distant lymph nodes than immature DCs did: 1.8% vs. 0.3% after intradermal injection and 19.3% (range, 0.4%–84%) vs. 10.2% (range, 0.5%–30%) after intranodal injection. Of further note, immature DCs resided at the periphery of the nodes, the marginal sinus, while mature DCs migrated deep into the T-cell areas.

### 4.3. Toxicity

Throughout all published trials it stands out that toxicity of RNA-modified DC vaccination is generally limited to low-grade adverse events with commonly occurring injection site reactions or more seldom systemic immune effects such as temperature elevation and flu-like symptoms. Very rarely there has been documentation of on-target immune-mediated bystander toxicity with low-grade vitiligo and grade 3–4 thrombocytopenia in the melanoma and AML setting, respectively [74,75,76,77]. Nearly all other cases of severe (grade 3–4) adverse immunological reactions were related to concomitant ipilimumab therapy in melanoma, while one case was due to the development of auto-antibodies against the GM-CSF component and not the DC in a specific vaccine for glioblastoma [78,79].

### 4.4. Immunological Responses

Immunological effects are based on a diverse array of ex vivo or in vitro immunological assays, most often relying on increased specific IFN-γ secretion by PBMCs or CD8+ T-cell responses as determined by IFN-γ release assays, MHC tetramer staining, or lytic activity. Some investigated in vivo immune responses using delayed-type hypersensitivity (DTH) skin tests.

Using those methods, nearly all studies report measurable or increased disease (antigen)-specific immune responses after DC vaccination in at least some of their patients (Table S1). Conversely, two studies found TAA-specific IFN-γ-secreting PBMCs and CD8+ T-cell proliferation before vaccination, which diminished following vaccination [80,81]. Other studies stated that they saw no tumor (antigen)-specific T cells after vaccination [82,83,84,85]. Additionally, one of the few RCTs showed no significant differences in HIV-1-specific IFN-γ-secretion by PBMCs after vaccination with HIV-1 mRNA-electroporated DCs compared to mock-electroporated DCs [86]. Two trials, in patients with peripheral or central nervous system tumors, described tumor-specific antibodies in some patients after vaccination, indicating a potential involvement of B cells/plasma cells, but more evidence is necessary to substantiate this [82,83]. A possible explanation for failing immunological effects could be the absence of pre-existing immunity specific for antigens presented by vaccine DCs, as several recent publications herald the importance of such pre-existing immunity to elicit antitumor immunity through DC vaccination [87,88,89]. Nonetheless, these studies all describe the use of neo-epitope peptide-loaded DCs, not RNA-loaded DCs. The contribution of pre-existing immunity to antitumor immune responses after TAA RNA-loaded DC vaccination should be further investigated.

Regardless of the extent of any immunological response, several larger studies (i.e., 30 or more patients each), including our own, provide robust evidence based on MHC-multimer staining that vaccination with DAA mRNA-loaded DCs can stimulate in vivo induction or expansion of polyclonal DAA epitope-specific T cells [78,90,91]. These results confirm those of an earlier study by Su et al., in which autologous tumor RNA-pulsed DCs generated previously undetectable polyclonal tumor and TAA-specific T cells in vivo based on ex vivo/in vitro IFN-γ enzyme-linked immunospot assay data [92]. Interestingly, in another study by the same group, continuing vaccination or further boosting seemed necessary to maintain the TAA-specific T-cell responses [93]. Besides the vaccination schedule, the administration route might potentially influence immune responses as well. In two phase I/II trials, one in metastatic castration-resistant prostate cancer and the other in advanced melanoma, totaling 41 patients, tumor-specific immune responses appeared more frequent after intradermal vaccination compared to intranodal vaccination [94,95]. In another trial in 15 patients with stage IIIc or IV melanoma, meaningful objective clinical responses were observed, including two complete remissions, two partial remissions, and four stable diseases, which the authors considered to be possible owing to the addition of intravenous on top of intradermal DC administration [96]. Altogether, it seems established that DAA RNA-loaded DC vaccination can induce disease-specific immune responses; however, the determinants influencing these responses remain unclear.

The in vivo immunological responses to RNA-loaded DC vaccination are not limited to disease-reactive immunity alone. When Su et al. detected increases in tumor and TAA-specific IFN-γ-secreting T cells in six out of seven evaluable patients, they simultaneously found low numbers of normal renal epithelium-specific IFN-γ-secreting T cells in all of those patients after DC vaccination [92]. Others demonstrated that tumor RNA-electroporated DC vaccination induced tumor-specific IFN-γ-secreting cells and cytotoxic T-lymphocyte clones, but also cells reactive against mock-electroporated DCs, indicating an immune response against both transfected and non-transfected antigens [94,95,97]. As mentioned before, studies in melanoma described the occurrence of vitiligo, an acquired depigmentation of the skin due to an immune-mediated destruction of melanocytes, in multiple patients after melanoma or melanoma-associated antigen RNA-electroporated DC vaccination [74,95]. All these findings suggest that DAA RNA-loaded DC vaccination may stimulate exaggerated on-target or off-target immune responses and even clinically relevant autoimmunity.

### 4.5. Clinical Responses

The clinical effects of RNA-modified DC vaccination are difficult to establish based on the available evidence. Thus far clinical trials were all (relatively) small phase I or phase II studies and often clinical outcome was only a secondary or even tertiary endpoint, if evaluated at all. Therefore, we looked at pooled data from these trials in HIV-1 and cancer. In HIV-1, there have been six trials including 108 patients, of which 86 underwent vaccination and 22 received placebo (Table S1). Three of these trials did not investigate clinical effects [86,98,99]. The remaining three trials involved 77 patients, including a placebo-controlled randomized phase IIb trial on 54 patients [100,101,102]. In these three trials that evaluated clinical efficacy, no clinical responses were detected. Specifically, there were no indications of efficacy compared to historical controls, no differences in viral RNA levels or CD4+ T-cell counts and no effect on the duration of antiretroviral therapy interruption [100,101,102].

In the setting of cancer, there are 25 trials that evaluated clinical responses (Table 2). Altogether, out of 437 vaccinated patients there are 62 complete responses/remissions (CRs; 14%), 27 partial responses/remissions (PRs; 6%), 96 stable diseases (SDs; 22%), three mixed responses (MRs; 0.7%), and 249 progressive diseases (PDs; 57%). The objective response rate (CR + PR) is 20% (89/424 patients) and the disease control or immune-related response rate (CR + PR + SD) is 42% (185/424 patients). It needs mentioning, however, that in most of these trials (1) patients were in CR at the start of DC vaccination and/or (2) DC vaccination was combined with other treatments including surgical resection, ablation, chemotherapy, radiotherapy, and other immunotherapy, which could all influence responses. Sifting through these data, we found five CRs and five PRs which appeared attributable to DC vaccination alone [82,90,91,96,103].

Equally noteworthy, several trials suggest meaningful immune-mediated clinical effects aside from objective responses. Recent work by others in glioblastoma and by our own group in AML demonstrated overall survival rates that compared favorably to matched historical controls or relevant large real-world database results [91,104]. Moreover, overall survival was correlated with increased TAA epitope-specific CD8+ T-cell frequencies after DC vaccination [91,105]. Our study with RNA-modified DC vaccination in AML patients in first CR with high risk of relapse further also suggested that vaccination can potentiate the response to subsequent therapy following relapse with a second CR rate and 5-year overall survival rate that was significantly higher than previously reported [91]. In summary, there is evidence that RNA-modified DC vaccination can induce objective clinical responses and survival benefit in cancer patients through induction of anti-cancer immunity.

## 5. Discussion

### 5.1. Shortcomings and Contributions of Published Clinical Trials

A first hurdle towards conclusions about the value of RNA-engineering DCs for therapeutic vaccination is the limited clinical data. A total of 52 clinical trials investigating RNA-modified DC vaccination were published, containing data on 696 different patients and averaging less than 20 patients per study. A major reason for these small numbers is the laborious and expensive production of these personalized vaccines. Another reason, probably related at least in part to the previous one, is that nearly all studies were driven by academia, with limited resources compared to pharmaceutical industry. The low objective response rates and the fact DC vaccination seems most efficacious in minimal residual disease, where clinical benefit may take longer to become apparent, are likely other factors explaining lack of commercial interest. Hence, only one “DC-like” vaccine is commercially available in the United States of America: the peptide-loaded antigen-presenting cell vaccine sipuleucel-T (Provenge™, Seal Beach, CA, USA) for patients with metastatic castration-resistant prostate cancer, marketed by Dendreon Pharmaceuticals LLC [117]. Of note, marketing authorization in Europe was withdrawn by Dendreon less than two years after receiving it, as they decided to permanently discontinue the marketing of the product for commercial reasons (EMA/303072/2015; 19 May 2015).

Systematic interpretation of immunological and clinical effects of RNA-modified DC vaccination in patients is prevented by an immense heterogeneity, which is apparent in vaccine preparation and administration, other interventions, comparators, immunological and clinical endpoints, as well as the reporting thereof. Just to illustrate, vaccination was carried out with 10^5^ to 10^8^ DCs on a weekly, biweekly, monthly, or bimonthly basis via the subcutaneous, intradermal, intranodal, or intravenous route, sometimes even combining approaches within the same trial. Furthermore, study populations were small and most often lacked a control group, thereby not allowing any firm conclusions or comparisons to be made and resulting in descriptive reporting. Upon in-depth combined review of both results and methodology, immunological and clinical responses are often ambiguous. Without a gold standard for immune monitoring following DC vaccination, the question arises whether the lack of consistent immunological responses is due to methodological shortcomings or the fact that responses are just not there. In specific settings of cancer, where self-antigens are moonlighting as TAAs, it could be argued that immune induction following TAA (m)RNA-loaded DC vaccination is insufficient to break tolerance. The reported clinical responses are nearly always equivocally attributable to DC vaccination, because most patients received one or more other treatments during the DC vaccination study period.

There are six RCTs on RNA-modified DC vaccination, of which one compared DC vaccination with chemotherapy versus chemotherapy alone, and three compared vaccination with RNA-loaded DC versus placebo or unloaded DC [81,86,101,105]. Two of these four trials did not evaluate clinical responses, while the other two showed no clinical benefit of RNA-loaded DC vaccination over chemotherapy alone or placebo. Despite all negative or ambiguous results, there is ample reliable evidence that vaccination with RNA-modified DCs is able to induce T cells specific for multiple defined or even undefined DAA epitopes in patients, demonstrating the potential of RNA-loaded DCs to generate a broad T-cell repertoire. Moreover, there are reports of unequivocal objective clinical responses and of favorable survival rates compared to retrospective controls following RNA-modified DC vaccination.

### 5.2. Prospects

While the degree of clinical benefit of RNA-modified DC vaccination remains obscure, research on all levels from in vitro over preclinical to clinical studies has yielded valuable insights that drive innovation of DC vaccination further and even beyond in vitro generated DCs. Two major novel approaches focus on (1) combinations that modulate the immune interaction microenvironment or (2) targeting antigen or immune-regulatory molecules directly to DCs in vivo.

Modulating the immune interaction microenvironment most often boils down to tackling tumor immunosuppression and evasion, which is one of the hallmarks of cancer [3]. Dannull et al. showed that selectively eliminating CD25+ regulatory T cells by a recombinant IL-2 diphtheria toxin conjugate before tumor RNA-loaded DC vaccination resulted in enhanced cytotoxic T lymphocyte responses in vivo [118]. A drawback, however, is that CD25 is also upregulated on other T cells upon activation, explaining why the recombinant IL-2 diphtheria toxin conjugate only led to increased T-cell stimulation when it was omitted during priming [118]. These days, much more interest goes out to blockade of the potent immune checkpoint inhibitors cytotoxic T-lymphocyte-associated protein 4 (CTLA-4) and programmed cell death protein 1 (PD-1) and its ligands PD-L1/2, which has already demonstrated remarkable clinical efficacy in different cancers as monotherapy and is increasingly being combined with DC vaccination [119]. One clinical trial by Wilgenhof et al. combined ipilimumab (an anti-CTLA-4 antibody) with melanoma-associated antigen RNA-loaded DC vaccination thus far [78]. Clinical results were impressive with an objective response rate (CR + PR) of 38.5% ([8 CR + 7 PR]/39) and disease control rate (CR + PR + SD) of 53.8% (21/39), which compares favorably to the results from the landmark phase 3 trial with ipilimumab monotherapy of 10.9% and 28.5%, respectively [78,120]. In contrast to directly targeting immunosuppression, Mitchell et al. explored another interesting strategy to boost responses at the level of the immune interaction microenvironment. They demonstrated that preconditioning DC vaccine sites with the strong recall antigens tetanus and diphtheria toxoid increased DC migration to lymph nodes and resulted in improved antitumor efficacy and longer survival in glioblastoma patients after TAA RNA-loaded DC vaccination [121].

Moving beyond the laborious process of in vitro DC vaccine generation, several techniques have already proven successful at targeting antigen or immune-regulatory molecules directly to DCs in vivo with relevant in vivo efficacy. The simplest concept is the systemic or local administration of naked RNA. Suzuki et al. demonstrated that intraperitoneal injection with CD40 siRNA leads to a decrease of allergic immune reactions and nasal symptoms in an OVA allergy mouse model, which was associated with impaired antigen presentation by DCs [122]. In different mouse models of cancer, studies showed that intranodal injection of naked mRNA was as effective as vaccination with mRNA-electroporated DCs [123]. Moreover, a review by the same group underscores that intradermal vaccination with naked TAA mRNA can induce clinical benefit, including objective responses, in melanoma and renal cell carcinoma patients [124]. Taking this further, more recent work by Sahin et al. showed that personalized RNA vaccines based on individual mutations and predicted neo-epitopes can result in vaccine-induced T-cell infiltration and neo-epitope-specific killing of autologous cancer cells in metastases, objective responses, and sustained progression-free survival in melanoma patients [125].

More complex approaches involve packaging or formulating the RNA to specifically achieve uptake by DCs. Singh et al. included IL-10 siRNA and antigen DNA dual-loaded microparticles into an injectable chemokine-carrying hydrogel, which recruited immature DCs to the injection site and induced a strong T-helper 1 and cytotoxic T-lymphocyte response protecting against subsequent tumor challenge in a lymphoma mouse model [126]. Others packaged antigen mRNA into lipoplexes which enter DCs through macropinocytosis, and found that such complexes target to DCs in vivo after intravenous administration, display intrinsic adjuvant activity via TLR signaling, and can induce tumor rejection in melanoma and lung cancer mouse models [127,128]. Alternatively, by formulating melanoma-associated antigen mRNA to target the DC phagocytosis antigen uptake receptor DEC-205 through fusion to an anti-DEC-205scFv antibody region, Birkholz et al. significantly increased MHC class II presentation compared to peptide pulsing or mRNA electroporation [129]. Although surpassing RNA modification, recent work by Cauwels et al. for targeting DCs in vivo deserves mention. They developed a mutated form of IFN-α2 coupled to a single domain antibody specific for the DC receptor Clec9A, which is up to 1000-fold more potent on DC target cells and yields strong antitumor immune responses resulting in complete tumor regression and long-lasting protection in melanoma, breast cancer, and lymphoma mouse models without significant toxicity [130]. This approach is excitingly promising, but of course targets all DCs irrespective of the antigens they present, while RNA modification allows to combine IFN-α and TAA expression in DCs [41].

## 6. Conclusions

From 2001 to date, 52 published clinical trials have reported on 696 different patients vaccinated with RNA-modified DCs. All trials were pilot or early phase studies and there are no phase 3 studies so far. Virtually all patients suffered from cancer or HIV-1 and were vaccinated with DAA RNA-loaded DCs, with the last 13 years exclusive use of mature DCs—as opposed to immature DCs before 2006—which were almost always loaded with RNA through electroporation. Unfortunately, heterogeneity in study design, methodology and endpoints, generally small population sizes without a control group, frequent combination with other treatment modalities during the study period, and the inherent complexity of immune-related responses in cancer resulted in ambiguity and prevent from reliably quantifying any clinical effects. Despite these shortcomings, clinical trials provided evidence that RNA-modified DC vaccination can induce objective clinical responses and survival benefit in cancer patients through stimulation of anti-cancer immunity, without significant toxicity. These promising observations continue to fuel efforts towards developing strategies to improve the clinical efficacy of DC vaccination. Besides the ongoing exploration of RNA modification of in vitro generated vaccine DCs, focus mainly lies on novel combination therapies to improve immune interaction and response after DC vaccination, as well as on approaches to target antigen or immune-regulatory molecules directly to DCs in vivo.

## Figures and Tables

**Table 1:** In vitro transcribed (IVT) RNA products investigated in RNA-modified dendritic cell vaccination clinical trials.

Category	Product/Target	*n* Vaccinated Subjects
Messenger RNA		
Disease-associated antigens		
Melanoma-associated	tyrosinase	144
	gp100	144
	Mage-A3	108
	Mage-C2	84
	MelanA/Mart-1	42
	Mage-A1	30
Other TAAs	hTERT	99
	survivin	69
	CEA	52
	Muc1	42
	WT1	38
	PSA	37
	p53	26
	PAP	21
	Hsp70	12
	FR-α	1
HIV-1	Nef	86
	Rev	76
	Gag	69
	Vpr	53
	Tat	23
CMV	pp65	42
Co-stimulatory molecules		
	CD70	68
	CD40L	68
Danger signals		
	cTLR4	68
**Small interfering RNA**		
	Three inducible iP subunits	5
	IDO	4

Abbreviations: CD, cluster of differentiation; CEA, carcinoembryonic antigen; CMV, cytomegalovirus; cTLR4, constitutively-active Toll-like receptor-4; FR-α, folate receptor-α; gp100, glycoprotein 100; HIV-1, human immunodeficiency virus-1; Hsp70, 70 kilodalton heat shock protein; hTERT, human telomerase reverse transcriptase; IDO, indoleamine 2-3-deoxygenase; iP, immunoproteasome; IVT, in vitro transcribed; Mage, melanoma-associated antigen; MelanA/Mart-1, melanoma antigen/melanoma antigen recognized by T cells 1; Muc1, Mucin 1, cell surface associated; RNA, ribonucleic acid; PAP, prostate acid phosphatase; PSA, prostate-specific antigen; TAA, tumor-associated antigen; WT1, Wilms’ tumor 1.

**Table 2:** Clinical responses following RNA-modified dendritic cell vaccination.

Trial Author [Ref]	Disease	CR	PR	SD	MR	PD	Total	Other Treatment	Remarks
		*n* (%)	*n* (%)	*n* (%)	*n* (%)	*n* (%)	*n*		
Khoury [77]	AML in CR + high relapse risk	11 (52)	-	-	-	10 (48)	21	-	Sustained CRs
Anguille [91]	AML in CR + high relapse risk	6 (21)	-	-	-	22 (79)	28	-	Four sustained CRs
Wilgenhof [78]	Stage III/IV melanoma	8 (21)	7 (18)	6 (15)	-	18 (46)	39	Ipilimumab	-
Maeda [106]	HCC	1 (8)	1 (8)	5 (42)	-	5 (42)	12	Resection/ablation	-
Shindo [107]	Pancreatic cancer	1 (2)	3 (7)	22 (52)	-	16 (38)	42	Gemcitabine	-
Dannull [103]	Stage IV melanoma	6 (50)	1 (8)	-	-	5 (42)	12	-	Five sustained CRs
Wilgenhof [96]	Stage III/IV melanoma	2 (13)	2 (13)	4 (27)	-	7 (47	15	-	-
Morse [108]	CEA+ metastatic cancer MRD CEA+ metastatic cancer	3 (25) -	- -	- 6 (25)	- -	9 (75) 18 (75)	12 24	- -	Sustained CRs -
Morse [109]	Pancreatic cancer	3 (100)	-	-	-	-	3	5-FU + RT, resection	Sustained CRs
Wilgenhof [74]	Stage III/IV melanoma	10 (33)	-	-	-	20 (67)	30	Resection +/− IFN	Sustained CRs
Aarntzen [90]	Stage III melanoma Stage IV melanoma	11 (42) -	- 1 (5)	- 5 (23)	- 1 (5)	15 (58) 15 (68)	26 22	- -	Sustained CRs -
Kongsted [81]	Progressive metastatic CRPC	-	1 (25)	2 (50)	-	1 (25)	4	Docetaxel	-
Kyte [75]	Stage IV melanoma	-	1 (3)	3 (10)	-	25 (86)	29	+/− IL-2	-
Amin [110]	mRCC	-	9 (43)	4 (19)	-	8 (38)	21	Resection, sunitinib	-
Caruso [82]	Relapsed CNS tumors	-	1 (14)	4 (57)	-	2 (29)	7	-	-
Borch [80]	Stage IV melanoma	-	-	9 (41)	-	13 (59)	22	CTX	-
Coosemans [111]	Uterine cancer	-	-	1 (17)	1 (17)	4 (67)	6	-	-
Hobo [112]	Stage II/III myeloma	-	-	5 (50)	-	5 (50)	10	-	-
Suso [113]	Pancreatic cancer	-	-	1 (100)	-	-	1	-	-
Markovic [84]	Stage IV melanoma	-	-	1 (17)	-	5 (83)	6	-	-
Mu [94]	Metastatic CRPC	-	-	11 (58)	-	8 (42)	19	-	SD based on PSA
Caruso [83]	Stage IV PNB	-	-	1 (9)	-	10 (91)	11	Resection, CRT	-
Rains [114]	Metastatic CRC	-	-	6 (50)	-	6 (50)	12	-	-
Sioud [115]	Stage IV melanoma	-	-	-	1 (100)	-	1	Prior ipilimumab, RT	-
Coosemans [116]	Ovarian cancer	-	-	-	-	2 (100)	2	-	-
**Total**		**62 (14)**	**27 (6)**	**96 (22)**	**3 (0.7)**	**249 (57)**	**437**		
**CR + PR**			**89 (20)**						
**CR + PR + SD**				**185 (42)**					

Abbreviations: 5-FU, 5-fluorouracil; AML, acute myeloid leukemia; CEA, carcinoembryonic antigen; CNS, central nervous system; CR, complete response/remission; CRC, colorectal cancer; CRT, chemoradiotherapy; CTX, cyclophosphamide; HCC, hepatocellular carcinoma; IFN, interferon; IL, interleukin; CRPC, metastatic castration-resistant prostate cancer; mRCC, metastatic renal cell carcinoma; MRD, minimal residual disease; PD, progressive disease; PNB, peripheral neuroblastoma; PR, partial response/remission; PSA, prostate-specific antigen; RT, radiotherapy; SD, stable disease.

## Supplementary Materials

The following are available online at https://www.mdpi.com/2072-6694/12/2/299/s1, Table S1: Clinical trials with RNA-modified dendritic cell vaccination.
