# Supplementary material for: Ribonucleic Acid Engineering of Dendritic Cells for Therapeutic Vaccination: Ready ‘N Able to Improve Clinical Outcome?
Source: Cancers (Basel). 2020 Jan 27;12(2):299. doi: 10.3390/cancers12020299 (PMC7072269; doi:10.3390/cancers12020299)
Supplement: Supplementary file 1 [file cancers-12-00299-s001.pdf]

Supplementary Materials

Ribonucleic Acid Engineering of Dendritic Cells for Therapeutic Vaccination: Ready 'N Able to Improve Clinical Outcome?

Yannick Willemen, Maarten Versteven, Marc Peeters, Zwi N. Berneman and Evelien L.J. Smits

Table S1. Clinical trials with RNA-modified dendritic cell vaccination.

First author, year [ref]<br>Trial design<br>Disease specifics	*n*	DC[a] + loading<br>RNA source<br>and product	Vaccine dose,<br>other therapies	Immunological results	Clinical results
**Stage III/IV melanoma**					
Kyte et al, 2016 [1]<br>Phase I/II 2-arm<br>Metastatic (stage IV) melanoma, except 1 stage III	31	mDC + EP<br>Tumor RNA<br>(autologous)	1–2 × 10^7 DC IN (*n* = 21) or ID (*n* = 10) −/+ IL-2 (*n* = 22 vs 9)	16/31 "immune responders"<br>14/28 ↑ tumor-specific PBMC proliferation (fresh or after in vitro restimulation)<br>12/28 weak-strong vaccine DTH reaction<br>More immune responders after ID vs IN vaccination (80 vs 38%) (includes data from Mu et al, 2005)	Median OS 10 m, significantly ↑ survival in immune responders vs non-responders<br>1/29 PR, 3/29 SD, 25/29 PD<br>2/31 rapid PD<br>1/22 vitiligo grade 1
Sioud et al, 2016 [2]<br>Case<br>Metastatic melanoma	1	mDC + EP<br>IVT mRNA<br>TAA (hTERT or survivin) + IVT siRNA IDO	5 × 10^6 DC (hTERT) + 5 × 10^6 DC (survivin) ID	↑ hTERT- and survivin-specific T-cell proliferation and PBMC IFN-γ secretion (after in vitro stimulation)<br>↑ survivin, MelanA and NY-ESO-1 epitope-specific CD8+ T cells (after in vitro stimulation)	MR, later PD
Borch et al, 2016 [3]<br>Phase I 1-arm<br>Metastatic melanoma in progression	26	mDC + EP<br>IVT mRNA<br>TAA (hTERT, survivin, p53)	CT + 5 × 10^6 DC ID	Ambiguous immune responses<br>6/17 TAA-specific IFN-γ-secreting PBMCs before or during vaccination<br>2/17 TAA-specific CD4+ T-cell proliferation during vaccination (after in vitro restimulation)<br>4/17 TAA-specific CD8+ T-cell proliferation before or during vaccination (after in vitro restimulation)	9/22 SD, 9/22 PD, 4/22 rapid PD<br>Median PFS 3.1 m<br>Median OS 10.4 m

Wilgenhof et al, 2016 [4]<br>Phase II 1-arm	39	mDC + EP<br>IVT mRNA<br>TriMix[b] +<br>TAA/LAMP<br>(tyr, Mage-A3,<br>Mage-C2 or<br>gp100)	4 × 10^6 (equal mixtures of TAA-loaded) DC ID + 2 × 10^7 (equal mixtures of TAA-loaded) DC IV + ipilimumab	↑ PB lymphocytes, eosinophils and monocytes<br>3/10 TAA epitope-specific CD8+ T cells (1 pre-existent)	8/39 CR, 7/39 PR, 6/39 SD, 18/39 PD<br>Median PFS 6.2 m<br>Median OS 13.6 m<br>24/39 completed induction, 8/39 discontinued due to toxicity, and 7/39 due to PD<br>14/39 grade 3–4 toxicity
Wilgenhof et al, 2015 [5]<br>Pilot 4-arm	30	mDC + EP<br>IVT mRNA<br>TAA/LAMP<br>(tyr, Mage-A1,<br>Mage-A3,<br>Mage-C2,<br>MelanA or<br>gp100)	Resection, 2.4 × 10^7 (equal mixtures of TAA-loaded) DC ID +/− adjuvant IFN-α2b	4/10 TAA-specific DTH-infiltrating cells	10/30 disease free, 20/30 relapsed<br>Median DFS 22 m<br>Median OS not reached after median FU 6.4 y<br>7/29 vitiligo
Dannull et al, 2013 [6]<br>Phase I 3-arm<br>Stage IV melanoma, 10/12 no evidence of disease after resection	12	mDC + EP<br>IVT mRNA<br>TAA (tyr,<br>Mage-A3,<br>MelanA, gp100)<br>+<br>IVT siRNA (3 iP<br>subunits,<br>control or none)	10^7 DC ID	12/12 ↑ TAA-specific IFN-γ-secreting CD4+ and CD8+ T cells (after in vitro restimulation)<br>2/6 ↑ autologous melanoma cell line-specific CTL lytic activity (both with iP-silenced DCs)	DC vs DC + control siRNA vs DC + IP siRNA 4 vs 3 vs 5 patients<br>1/2 CR and 1/2 transient PR (both with iP-silenced DCs), 5/10 sustained CR<br>6/11 remained disease-free at median FU 35 m
Wilgenhof et al, 2013 [7]<br>Phase IB 4-arm	15	mDC + EP<br>IVT mRNA<br>TriMix[a] +<br>TAA/LAMP<br>(tyr, Mage-A3,<br>Mage-C2 or<br>gp100)	20/12/4/0 × 10^6 DC ID + 4/12/20/24 × 10^6 DC IV (equal mixtures of TAA-loaded DC)	6/10 TAA epitope-specific DTH-infiltrating CD8+ T cells<br>5/12 TAA epitope-specific DTH-infiltrating CD4+ T cells	2/15 CR, 2/15 PR, 4/15 SD<br>Median PFS 5 m<br>Median OS 14 m
Aarntzen et al, 2012 [8]<br>Phase I(/II) 1-arm	48	mDC + EP<br>IVT mRNA<br>TAA (tyr,<br>gp100)	12 × 10^6 DC + KLH IN	6/45 TAA epitope-specific PB CD8+ T cells after vaccination (3/6 also before)<br>4/15 TAA epitope-specific PB CD4+ T cells after vaccination (1/4 also before)<br>28/45 TAA epitope-specific DTH-infiltrating CD8+ T cells	15/26 stage III relapsed, median DFS 34.3 m<br>3/48 rapid PD, 1/19 PR, 1/19 MR, 5/19 SD, trend for improved PFS and OS with vs without TAA epitope-specific CD8+ T cells (8.1 vs 2.8 m and 24.1 vs 11.0 m, resp.)

Wilgenhof et al, 2011 [9] (30/35 see Wilgenhof et al, 2015 [5]) Phase I 1-arm	5	mDC + EP IVT mRNA TriMix[a] + TAA/LAMP (tyr, Mage-A3, Mage-C2 or gp100)	17–66 × 10^6 (avg 43 × 10^6; equal mixtures of TAA-loaded) DC ID, IFN-α2b (29/32)	12/21 vaccine-specific DTH-infiltrating CD8+ T cells, 9/21 specific for > 1 TAA	9/15 relapsed, median DFS 23 m (n = 15) 11/20 SD, 9/20 PD, median PFS 3.1 m, median OS 15.1 m 32/35 4 vaccines feasible
Markovic et al, 2006 [10] Phase I 1-arm Metastatic melanoma	6	mDC + EP Tumor RNA (autologous)	5 × 10^6 DC SC	0/6 tumor-specific T cells	No objective responses 1/6 SD, 5/6 PD
			**Human immunodeficiency virus-1**		
Gay et al, 2018 [11] Retroviruses 1-arm sub-study ≥ 6 m ART + viral RNA < 50 copies/mL, nadir CD4+ T cells ≥ 200/mm³	6	mDC + EP IVT mRNA autologous viral HIV-1 (Gag, Vpr, Rev and Nef), CD40L	1.2 × 10^7 DC (+/- mRNA) ID, ATI + booster DC	6/6 increase HIV-1-specific CD28+/CD45RA- CD8+ EM CTLs	No sustained ART interruption Expansion of CD8+ effector T cells ~ longer time to viral rebound
Jacobson et al, 2016 [12] Phase IIb 2-arm RCT 2:1 ≥ 3 m viral RNA ≤ 200 copies/mL and < 50 copies/mL at screening, CD4+ T cells ≥ 450/mm³	54	mDC + EP IVT mRNA autologous viral HIV-1 (Gag, Vpr, Rev and Nef), CD40L	10^7 DC ID (n = 37) or placebo (n = 17), ATI + booster DC	↑ functional (+/- vaccine-specific) CD28+ CD45RA- CTLs after DCs vs placebo	No differences in viral RNA levels after placebo vs DCs No differences in CD4+ T-cell counts after placebo vs DCs
Gandhi et al, 2016 [13] Phase I 2-arm RCT 2:1 ≥ 1 y ART + CD4+ T cells ≥ 200/mm³, ≥ 6 m viral RNA < 50 copies/mL	15	mDC + EP IVT mRNA HIV-1/LAMP (Gag and Nef)	5–15 × 10^6 DC +/- mRNA (n = 10 vs 5) ID + 1.5–6 × 10^6 DC + KLH ID	No significant differences in Gag- or Nef-specific IFN-γ-secreting PBMCs after mRNA-EP DCs vs mock-EP DCs ↑ KLH-specific CD4+ and CD8+ T-cell proliferation	Not evaluated
Van Gulck et al, 2012 [14] Phase I/II 1-arm Stable under ART	6	mDC + EP IVT mRNA HIV-1/LAMP (Gag or chimeric Tat-Rev-Nef)	5 × 10^6 DC SC + 5 × 10^6 DC ID, ATI	6/6 ↑ and 5/6 broadened Gag-specific PBMC responses 4/5 ↑ CD8+ T-cell mediated viral suppression in vitro	Not evaluated

Allard et al, 2012 [15]<br>Phase I/II 1-arm<br>≥ 3 m ART + viral RNA ≤ 50 copies/mL + CD4+ T cells ≥ 500/mm³)	17	mDC + EP<br>IVT mRNA<br>HIV-1/LAMP (Tat, Rev or Nef)	5 × 10^6 DC SC + 5 × 10^6 DC ID, ATI	17/33 ↑ HIV-1-specific PBMC responses	No indication of efficacy compared to historical controls<br>11/17 resumed ART < 96 w after vaccination (median time to resumption 50 w)<br>6/17 remained off ART ≥ 96 w after vaccination
Routy et al, 2010 [16]<br>Phase I 1-arm<br>Viral RNA ≤ 200 copies/mL, ≥ 12 w ART	10	mDC + EP<br>IVT mRNA<br>autologous viral HIV-1 (Gag, Vpr, Rev and Nef), CD40L	10^7 DC ID	7/9 ↑ HIV-1-specific CD8+ T-cell proliferation after in vitro restimulation	Not evaluated<br>10/10 4 vaccines feasible
**Prostate cancer +/- metastases**					
Kongsted et al, 2017 [17]<br>Phase II 2-arm RCT 1:1<br>mCRPC in progression	43	mDC + EP<br>IVT mRNA<br>TAA (hTERT, survivin, PSA, PAP)	Docetaxel −/+ 5 × 10^6 DC ID	Ambiguous immune responses<br>9/18 TAA-specific IFN-γ-secreting PBMCs before (*n* = 6) or during (*n* = 8) vaccination	Docetaxel -/+ DC 19 vs 21 evaluable patients<br>3/7 PR, 4/7 SD on docetaxel alone vs 1/4 PR, 2/4 SD, 1/4 PD on combination therapy<br>No difference in PSA response rates, median<br>PFS 5.5 vs 5.7 m (ns) or DSS 21.9 vs 25.1 m (ns)
Mu et al, 2005 [18]<br>(see also Kyte et al, 2016 [1] for ID vs IN)<br>Phase I 2-arm<br>CRPC +/- bone M+	20	mDC + EP<br>Tumor RNA (3 PC cell lines)	2 × 10^7 DC IN or ID	10/19 tumor-specific IFN-γ-secreting PBMCs	11/19 SD (based on PSA), 8/19 PD<br>13/19 slower PSA increase (log slope pre vs post)<br>1/20 rapid PD
Su et al, 2005 [19]<br>Phase I 2-arm rdm dose-escalation<br>mPC	20	mDC + EP<br>IVT mRNA<br>TAA (hTERT) +/− LAMP-1	10^7 DC ID	11/12 induction of hTERT-specific CD8+ T cells<br>9/12 induction of hTERT-specific CD4+ T cells (more with LAMP-1)	No objective responses<br>↑ PSA doubling time (*n* = 5)
Heiser et al, 2002 [20]<br>Phase I 1-arm dose-escalation<br>mPC	16	iDC + PP<br>IVT mRNA<br>TAA (PSA)	10^7, 3 × 10^7 or 5 × 10^7 DC IV + 10^7 DC ID	8/8 ↑ PSA-specific IFN-γ-secreting PBMCs	Insufficient/ambiguous data<br>1/7 PSA decrease, 5/7 slower PSA increase (log slope pre vs post), 1/7 unaffected PSA increase<br>2/16 rapid PD, 1/16 sepsis, 2/16 skeletal PD, 4/16 intake of PSA-affecting compounds
**Acute myeloid leukemia in complete remission**					

Anguille et al, 2017 [21]<br>Phase II 3-arm<br>High relapse risk	30	mDC + EP<br>IVT mRNA<br>TAA (WT1) +/−<br>LAMP +/−<br>codon opt	5×10^6, 10^7 or 2×10^7 DC + KLH ID	6/12 ↑ WT1 epitope-specific CD8+ T cells	9/30 molecular remission (2 PR to CR, 4 later relapsed), 4/30 stable MRD<br>6/30 sustained CR1, 22/29 (1 did not reach CR1) relapsed, 1/29 lung cancer<br>Median OS 99.4 m
Khoury et al, 2017 [22]<br>Phase II 1-arm<br>CR1 + high relapse risk or CR2 > 6 m after CR1	21	mDC + EP<br>IVT mRNA<br>TAA (hTERT)<br>+/− LAMP-1	10^7 DC ID	11/19 TAA-specific IFN-γ-secreting PBMCs	2/21 early relapse<br>11/19 sustained CR, 8/19 relapsed (median FU 52 m)<br>3/21 (possible) treatment-related AE grade ≥ 3, incl. 1/21 ITP grade 4
			**Metastatic renal cell carcinoma**		
Amin et al, 2015 [23]<br>Phase II 1-arm	25	mDC + EP<br>Tumor RNA<br>(autologous)	Resection, sunitinib + 1.2×10^7 DC ID	10/14 ↑ functional CD28+ CD45RA- CTLs, correlated with duration of survival	9/21 PR, 4/21 SD, 8/21 PD<br>Median PFS 11.2 m<br>Median OS 30.2 m<br>10/22 subsequent therapy<br>1/25 rapid PD
Dannull et al, 2005 [24]<br>Phase I 2-arm RCT<br>1 ovarian	11	mDC + EP<br>Tumor RNA<br>(autologous)	10^7 DC ID +/− $DAB_{389}IL\text{-}2^c$ IV (4 d prior)	9/10 ↑ tumor-specific CD8+ (higher after DAB389IL-2, *n* = 6) and CD4+ T cells<br>7/7 ↓ CD4+/CD25high Tregs after DAB389IL-2	Not evaluated
Su et al, 2003 [25]<br>Phase I 1-arm dose-escalation	15	iDC + PP<br>Tumor RNA<br>(autologous)	Nephrectomy, 10^7, 3×10^7 or 5×10^7 DC IV + 10^7 DC ID	6/7 ↑ tumor RNA-specific IFN-γ-secreting T cells<br>7/7 low numbers of benign renal tissue-specific IFN-γ-secreting T cells<br>Small increases in defined TAA-specific IFN-γ-secreting T cells	Insufficient/ambiguous data<br>Mean OS 19.8 m<br>5/15 rapid PD
			**Pancreatic cancer**		
Shindo et al, 2014 [26]<br>Retrospective<br>Unresectable or recurrent pancreatic cancer	42	mDC + EP<br>IVT mRNA<br>TAA (Muc1)	CT + 0.04–4 × 10^7 (avg 1.8×10^7) DC ID + 1–12×10^8 (avg 6×10^8) CTL IV	Mean increase in Muc1-specific IFN-γ-secreting PBMCs (*n* = 6)	1/42 CR, 3/42 PR, 22/42 SD, 16/42 PD<br>Median OS 13.9 m
Suso et al, 2011 [27]<br>Case<br>Pancreatic AC + LN M+	1	mDC + EP<br>IVT mRNA<br>TAA (hTERT)	CT, 5×10^6 DC ID	1/1 ↑ hTERT epitope-specific CD8+ T cells	1/1 SD/PR (42 m FU)
Morse et al, 2002 [28]<br>Phase I 1-arm	3	iDC + PP<br>IVT mRNA<br>TAA (CEA)	Neoadjuvant CRT (2/3), complete	Insufficient data	3/3 sustained CR (3.75, 3.75 and 4 y)

CEA+ pancreatic papillary mucinous (1/3) or AC (2/3)			resection, 10^7 DC ID		
**Glioblastoma**					
Reap et al, 2018 [29]<br>Pilot 2-arm RCT 1:1	17	mat [iDC + EP]<br>IVT mRNA<br>CMV<br>pp65/LAMP	Resection, CRT, CT, 3 × 10^7/kg pp65-specific T cells + 2 × 10^7 DC (n = 9) or placebo (n = 8) ID	↑ polyfunctional (IFN-γ^+ TNF-α^+ CCL3$^+$) pp65-specific CD8$^+$ T cells in 4/8 (DC) vs 1/7 (placebo) patients	Placebo vs DC 7 vs 8 efficacy evaluable patients<br>No severe AEs<br>Fold change of polyfunctional pp65-specific CD8$^+$ T cells correlated with OS after DC
Batich et al, 2017 [30]<br>Phase I 1-arm	14	mat [iDC + PP]<br>IVT mRNA<br>CMV<br>pp65/LAMP	Resection, CRT, CT + 2 × 10^7 DC ID + 150 μg GM-CSF	11/14 evaluable patients<br>10/11 CMV-specific IFN-γ-secreting PBMCs<br>↑ pp65 epitope-specific CD8+ T cells	11/14 evaluable patients<br>Median PFS 25.3 m<br>Median OS 41.1 m, 30 m gain vs historical controls<br>1/11 grade 3 allergic reaction to GM-CSF
Mitchell et al, 2015 [31]<br>Phase I 2-arm RCT 1:1	13	mat [iDC + PP]<br>IVT mRNA<br>CMV<br>pp65/LAMP	Resection, CRT, unpulsed DCs vs Td, CT + 2 × 10^7 DC ID +/− autologous lymphocytes	↑ pp65-specific IFN-γ-secreting PBMCs and DC migration to LNs correlated with OS	1/13 rapid PD (before vaccination)<br>↑ pp65-specific IFN-γ-secreting PBMCs correlated with OS Unpulsed DC vs Td preconditioning median PFS and OS 10.8 m and 18.5 m vs median PFS and OS not reached
Vik-Mo et al, 2013 [32]<br>Phase I 1-arm (vs historical controls)<br>No need for cortico-steroids	7	mDC + EP<br>Tumor RNA (autologous) + IVT mRNA<br>TAA (hTERT or survivin) in 5/7	Resection, CRT, 10^7 DC ID	7/7 ↑ lymphocyte proliferation after in vitro stimulation with either autologous tumor cell lysate, hTERT or survivin at some point during vaccination vs baseline	5/7 relapsed<br>Median PFS 22.8 m<br>Median OS 24.9 m (from resection)
**CEA+ metastatic cancer**					
Morse et al, 2003 [33]<br>Phase I 1-arm dose-escalation	31	iDC + PP<br>IVT mRNA<br>TAA (CEA)	10^7 DC IV, × 10^7 DC IV + 10^6 DC ID, 10^8 DC IV + 10^6 DC ID −/+ IL-2	Insufficient/ambiguous data	Insufficient/ambiguous data<br>6/24 SD, 18/24 PD, median OS 15.7 m<br>6/14 high dose unfeasible
Phase II 1-arm<br>No evidence of disease after surgery (MRD), 11/13 CRC with resected liver M+	13		3 × 10^7 DC IV + 10^6 DC ID		3/12 sustained CR<br>9/12 relapsed, median DFS 4.0 m
**Colorectal cancer +/- metastases**					

Lesterhuis et al, 2010 [34]<br>Phase I 1-arm<br>CRC + resectable liver M+	5	mDC + EP<br>IVT mRNA<br>TAA (CEA)	5×10^6 DC + KLH ID + 7–17×10^6 (avg 11 × 10^6) DC + KLH IV	0/5 CEA-specific DTH-infiltrating CD8+ T cells	4/5 median PFS 26 m after resection
Rains et al, 2001 [35]<br>Phase I/II 1-arm<br>Metastatic CRC	15	iDC + PP<br>Tumor RNA<br>(autologous)	avg 0.43–2.4×10^6 DC + KLH IV	11/13 positive KLH skin test	6/12 SD, 6/12 PD<br>7/13 CEA decrease<br>2/13 CEA slower rise
**Stage II/III multiple myeloma**					
Hobo et al, 2013 [36]<br>Phase I 1-arm<br>CR/PR after prior therapy	12	mDC + EP<br>IVT mRNA<br>TAA (Mage-A3, survivin, BCMA)	4–11×10^6 (median 8 × 10^6) DC + KLH ID + 5–22 × 10^6 (median 1.5 × 10^7) DC + KLH IV	6/12 DTH reaction with 2/6 TAA-specific IFN-γ-secreting DTH-infiltrating CD4+ and CD8+ T cells	10/12 alive at median FU 25 m after vaccination, 5/10 SD, 5/10 PD
**Hepatocellular carcinoma**					
Maeda et al, 2015 [37]<br>Phase I 3-arm<br>HCV-related HCC<br>(untreated, only SD)	12	mDC + EP<br>IVT mRNA<br>Hsp70	Resection or ablation, 3 × 10^7, 2 × 10^7 or 3 × 10^7 DC ID	Mean increase in Hsp70-specific IFN-γ-secreting PBMCs (ns)	1/12 CR, 1/12 PR (conversion to CR), 5/12 SD, 5/12 PD<br>Grade 3 liver abscess
**Stage IV adrenal or retroperitoneal neuroblastoma**					
Caruso et al, 2005 [38]<br>Phase I 1-arm dose-escalation	11	iDC + PP<br>Tumor RNA<br>(autologous)	CT, apheresis, CT, CT, resection, RT, CT, HSCT, 5 × 10^6 DC IV + 5 × 10^6 DC ID	0/5 tumor-specific T cells<br>2/3 ↑ tumor-specific antibodies	7/11 vaccinated (3/11 rapid PD, 1/11 insufficient DC)<br>1/11 SD, 10/11 PD and dead, median PFS 14 m, median OS 19 m
**Relapsed central nervous system tumors**					
Caruso et al, 2004 [39]<br>Phase I 1-arm dose-escalation<br>Low and high grade	9	iDC + PP<br>Tumor RNA<br>(autologous)	5 × 10^6 DC IV + 5 × 10^6 DC ID	0/7 tumor-specific T cells<br>2/5 ↑ tumor-specific antibodies	1/7 PR, 4/7 SD and 2/7 PD after 8 w<br>2/5 SD and 3/5 PD after 18 w<br>1/9 rapid PD, 1/9 insufficient RNA
**Cytomegalovirus**					
Van Craenenbroeck et al, 2015 [40]<br>Pilot<br>Preventive	7	mDC + EP<br>IVT mRNA<br>CMV pp65	1 × 10^7 or 1 × 10^5 DC ID	3/4 pp65-specific IFN-γ-secreting PBMCs (after in vitro restimulation)<br>0/4 CMV seroconversion	1/4 grade 2 GI GVHD
**Ovarian cancer**					

Coosemans et al, 2013 [41] Cases	2	mDC + EP IVT mRNA TAA (WT1)	Multiple CT, 7–61 × 10^6 (avg 21 × 10^6) DC ID + topical imiquimod	1/2 ↑ WT1 epitope-specific CD8+ T cells	2/2 PD
Sioud et al, 2013 [42] Phase I 1-arm (pilot) FIGO stage IIIc (2/4), uterine M+, peritoneal	4	mDC + EP IVT mRNA TAA (hTERT or survivin) + IVT siRNA IDO	5 × 10^6 DC (hTERT) + 5 × 10^6 DC (survivin) ID	↑ hTERT-(4/4) and survivin-specific (3/4) PBMC proliferation (after in vitro stimulation)	Insufficient/ambiguous data
Hernando et al, 2007 [43] Case FIGO stage IIIc	1	mDC + EP IVT mRNA TAA (FR-α)	2 × [CT, debulking], CT, LN dissection, 2×10^6 and 17–25×10^6 DC IN	↑ tumor-specific CD8+ and CD4+ T cells	PR at 16 m ↓ CA-125 at 4 w (640 to 60 U/mL)
			**Uterine cancer**		
Coosemans et al, 2013 [44] Phase I/II 1-arm (pilot)	6	mDC + EP IVT mRNA TAA (WT1)	Multiple CT, 1–32 × 10^6 (avg 6–22 × 10^6) DC ID + topical imiquimod	2/4 ↑ WT1 epitope-specific CD8+ T cells	1/6 MR, 1/6 SD, 4/6 PD
			**Early reports on patients for which longer follow-up data have been published**		
Van Nuffel et al, 2012 [45] (see Wilgenhof et al, 2013 [7]) Case Stage IV-M1c melanoma	1	mDC + EP IVT mRNA TriMix[a] + TAA/LAMP (tyr, Mage-A3, Mage-C2 or gp100)	CT, 11 × 10^6 DC ID + 20 × 10^6 DC IV (equal mixtures of TAA-loaded DC)	Expansion of multiple different TAA epitope-specific CD8+ T cells	SD after 8 w, PR after 16 w
Schuurhuis et al, 2009 [46] (see Aarntzen et al, 2012 [8]) Phase I 1-arm Tyr and gp100-expressing stage III melanoma	11	mDC + EP IVT mRNA TAA (tyr or gp100)	Unspecified number of DC + KLH IN, LN dissection	7/11 TAA-specific DTH-infiltrating CD8+ T cells	Not evaluated

Kyte et al, 2006 [47] (see Kyte et al, 2016 [1]) Phase I/II 2-arm Metastatic melanoma, 1 stage III	24	mDC + EP Tumor RNA (autologous)	2 × 10^7 DC IN or ID	10/19 ↑ tumor-specific PBMC proliferation (fresh or after in vitro restimulation) and/or DTH reaction	2/20 SD, 18/20 PD Mean OS 12.3 m (n = 22) 1/22 vitiligo grade 1 2/24 rapid PD
Van Tendeloo et al, 2010 [48] (see Anguille et al, 2017 [21]) Phase I/II 1-arm AML MRD	10	mDC + EP IVT mRNA TAA (WT1)	5 × 10^6, 10^7 or 2 × 10^7 DC + KLH ID	2/5 WT1 epitope-specific CD8+ T cells ↑ WT1-specific IFN-γ-secreting CD8+ T cells (after in vitro restimulation)	2/2 conversion of PR to CR 4/7 ↓ blood WT1 mRNA levels 6/9 relapsed, 3/10 sustained CR 1/10 thrombocytopenia grade 3
Van Driessche et al, 2009 [49] (see Anguille et al, 2017 [21]) Phase I 1-arm dose-escalation AML MRD, 1 MDS	10	mDC + EP IVT mRNA TAA (WT1)	5 × 10^6, 10^7 or 2 × 10^7 DC + KLH ID	Not evaluated	Not evaluated 7/10 4 vaccines at desired dose feasible
Mitchell et al, 2015 [50] (see Batich et al, 2017 [30]) Case Glioblastoma	1	mat [iDC + PP] IVT mRNA CMV pp65/LAMP	Resection, CRT, CT + 2 × 10^7 DC ID + 800 U GM-CSF	Anti-GM-CSF IgE, IgM, IgG	Grade 3 allergic reaction (hives, rash, headache, confusion, flushes, conjunctivitis) to GM-CSF
Nair et al, 2002 [51] (see Morse et al, 2003 [33]) Case CEA+ metastatic ACUP	1	iDC + PP Tumor RNA (autologous) IVT mRNA TAA (CEA; 6 m before in other trial)	3 × 10^7 DC IV + 10^6 DC ID	↑ specific tumor RNA-loaded DC target lysis by PBMC	No clinical response (continued PD)
Coosemans et al, 2010 [52] (see Coosemans et al, 2013 [44]) Case Stage IV uterine cancer	1	mDC + EP IVT mRNA TAA (WT1)	CT, debulking, multiple CT, 6–8.8 × 10^6 DC ID + topical imiquimod	1/1 ↑ WT1 epitope-specific CD8+ T cells	1/1 PD

[a] All immature (iDC), mature (mDC) and matured immature (mat [iDC + x]) dendritic cells were monocyte-derived. [b] TriMix mRNA is a mixture of CD40L, CD70 and constitutively-active Toll-like receptor-4 mRNA. [c] Conjugate of diphtheria toxin to recombinant IL-2.

Abbreviations: ↑: increased; ↓, decreased; AC, adenocarcinoma; ACUP, adenocarcinoma of unknown primary; AE, adverse event; AML, acute myeloid leukemia; ART, antiretroviral therapy; ATI, analytical treatment interruption; avg, average; BCMA, B-cell maturation antigen; CEA, carcinoembryonic antigen; CMV, cytomegalovirus; CNS, central nervous system; CR, complete response/remission; CRC, colorectal cancer; CRT, chemoradiotherapy; CT, chemotherapy; CTL, cytotoxic T lymphocyte; DC, dendritic cell; DFS, disease-free survival; DSS, disease-specific survival; DTH, delayed-type hypersensitivity skin test; ELISpot, enzyme-linked immunospot; EM, effector/memory; EP, electroporation; FIGO, International Federation of Gynecology and Obstetrics (Fédération Internationale de Gynécologie et d'Obstétrique); FR-α, folate receptor-α; FU, follow up; GI, gastrointestinal; GVHD, graft-versus-host disease; gp100, glycoprotein 100; HCC, hepatocellular carcinoma; HCV, hepatitis C virus; HIV, human immunodeficiency virus; HSCT, hematopoietic stem-cell transplantation; Hsp70, 70 kilodalton heat shock protein; hTERT, human telomerase reverse transcriptase; ID, intradermal; IDO, indoleamine 2–3-deoxygenase; IFN, interferon; IL, interleukin; IN, intranodal; iP, inducible immunoproteasome; IT, immunotherapy; ITP, idiopathic thrombocytopenic purpura; IV, intravenous; IVT, in vitro transcribed; KLH, keyhole limpet hemocyanin; LAMP, lysosome-associated membrane protein; LE, life expectancy; LF, lipofection; LN, lymph node; M+, metastasis; Mage, melanoma-associated antigen; mCRPC, metastatic castration-resistant prostate cancer; MelanA, melanoma antigen; mPC, metastatic prostate cancer; MR, mixed response; MRD, minimal residual disease; Muc1, Mucin 1, cell surface associated; ns, not significant according to predefined statistics; OS, overall survival; PAP, prostate acid phosphatase; PB, peripheral blood; PBMC, peripheral blood mononuclear cells; PD, progressive disease; PFS, progression-free survival; PP, passive pulsing; PR, partial response/remission; PSA, prostate-specific antigen; RCT, randomized controlled trial; rdm, randomized; RT, radiotherapy; SC, subcutaneous; SD, stable disease; TAA, tumor-associated antigen; Td, tetanus/diphtheria toxoid; tyr, tyrosinase; WT1, Wilms' tumor 1.

Reference

1. Kyte, J.A.; Aamdal, S.; Dueland, S.; Sæbøe-Larsen, S.; Inderberg, E.M.; Madsbu, U.E.; Skovlund, E.; Gaudernack, G.; Kvalheim, G. Immune response and long-term clinical outcome in advanced melanoma patients vaccinated with tumor-mRNA-transfected dendritic cells. *Oncoimmunology* **2016**, *5*: e1232237.
2. Sioud, M.; Nyakas, M.; Sæbøe-Larssen, S.; Mobergslien, A.; Aamdal, S.; Kvalheim, G. Diversification of Antitumour Immunity in a Patient with Metastatic Melanoma Treated with Ipilimumab and an IDO-Silenced Dendritic Cell Vaccine. *Case Rep. Med.* **2016**. doi: 10.1155/2016/9639585.
3. Borch, T.H.; Engell-Noerregaard, L.; Zeeberg Iversen, T.; Ellebaek, E.; Met, Ö.; Hansen, M.; Andersen, M.H.; Thor Straten, P.; Svane, I.M. mRNA-transfected dendritic cell vaccine in combination with metronomic cyclophosphamide as treatment for patients with advanced malignant melanoma. *Oncoimmunology* **2016**. 5, e1207842.
4. Wilgenhof, S.; Corthals, J.; Heirman, C.; van Baren, N.; Lucas, S.; Kvistborg, P.; Thielemans, K.; Neyns, B. Phase II Study of Autologous Monocyte-Derived mRNA Electroporated Dendritic Cells (TriMixDC-MEL) Plus Ipilimumab in Patients With Pretreated Advanced Melanoma. *J. Clin. Oncol.* **2016**, *34*, 1330–1338.
5. Wilgenhof, S.; Corthals, J.; Van Nuffel, A.M.; Benteyn, D.; Heirman, C.; Bonehill, A.; Thielemans, K.; Neyns, B. Long-term clinical outcome of melanoma patients treated with messenger RNA-electroporated dendritic cell therapy following complete resection of metastases. *Cancer Immunol. Immunother*. **2015**, *64*, 381–388.
6. Dannull, J.; Haley, N.R.; Archer, G.; Nair, S.; Boczkowski, D.; Harper, M.; De Rosa, N.; Pickett, N.; Mosca, P.J.; Burchette, J.; et al. Melanoma immunotherapy using mature DCs expressing the constitutive proteasome. *J. Clin. Invest.* **2013,** *123*, 3135–3145.
7. Wilgenhof, S.; Van Nuffel, A.M.; Benteyn, D.; Corthals, J.; Aerts, C.; Heirman, C.; Van Riet, I.; Bonehill, A.; Thielemans, K.; Neyns, B.; et al. A phase IB study on intravenous synthetic mRNA electroporated dendritic cell immunotherapy in pretreated advanced melanoma patients. *Ann. Oncol.* **2013**, *24*, 2686–2693.
8. Aarntzen, E.H.; Schreibelt, G.; Bol, K.; Lesterhuis, W.J.; Croockewit, A.J.; de Wilt, J.H.; van Rossum, M.M.; Blokx, W.A.; Jacobs, J.F.; Duiveman-de Boer, T.; et al. Vaccination with mRNA-electroporated dendritic cells induces robust tumor antigen-specific CD4+ and CD8+ T cells responses in stage III and IV melanoma patients. *Clin. Cancer Res.* 2012, 18, 5460–5470.
9. Wilgenhof, S.; Van Nuffel, A.M.; Corthals, J.; Heirman, C.; Tuyaerts, S.; Benteyn, D.; De Coninck, A.; Van Riet, I.; Verfaillie, G.; Vandeloo, J.; et al. Therapeutic vaccination with an autologous mRNA electroporated dendritic cell vaccine in patients with advanced melanoma. *J. Immunother.* **2011**. *34*, 448–456.

10. Markovic, S.N.; Dietz, A.B.; Greiner, C.W.; Maas, M.L.; Butler, G.W.; Padley, D.J.; Bulur, P.A.; Allred, J.B.; Creagan, E,T.; Ingle, J.N.; et al. Preparing clinical-grade myeloid dendritic cells by electroporation-mediated transfection of in vitro amplified tumor-derived mRNA and safety testing in stage IV malignant melanoma. *J. Transl. Med.* **2006**, *4*, 35.
11. Gay, C.L.; DeBenedette, M.A.; Tcherepanova, I.Y.; Gamble, A.; Lewis, W.E.; Cope, A.B.; Kuruc, J.D.; McGee, K.S.; Kearney, M.F.; Coffin, J.M.; et al. Immunogenicity of AGS-004 Dendritic Cell Therapy in Patients Treated During Acute HIV Infection. *AIDS Res. Hum. Retroviruses* **2018**, *34*, 111–122.
12. Jacobson, J.M.; Routy, J.P.; Welles, S.; DeBenedette, M.; Tcherepanova, I.; Angel, J.B.; Asmuth, D.M.; Stein, D.K.; Baril, J.G.; McKellar, M.; et al. Dendritic Cell Immunotherapy for HIV-1 Infection Using Autologous HIV-1 RNA: A Randomized, Double-Blind, Placebo-Controlled Clinical Trial. *J. Acquir. Immune Defic. Syndr.* **2016**, *72*, 31–38.
13. Gandhi, R.T.; Kwon, D.S.; Macklin, E.A.; Shopis, J.R.; McLean, A.P.; McBrine, N.; Flynn, T.; Peter, L.; Sbrolla, A.; Kaufmann, D.E.; et al. Immunization of HIV-1-Infected Persons With Autologous Dendritic Cells Transfected With mRNA Encoding HIV-1 Gag and Nef: Results of a Randomized, Placebo-Controlled Clinical Trial. *J. Acquir. Immune Defic. Syndr.* **2016**, *71*, 246–253.
14. Van Gulck, E.; Vlieghe, E.; Vekemans, M.; Van Tendeloo, V.F.; Van De Velde, A.; Smits, E.; Anguille, S.; Cools, N.; Goossens, H.; Mertens, L.; et al. mRNA-based dendritic cell vaccination induces potent antiviral T-cell responses in HIV-1-infected patients. *AIDS* **2012**, *26*, F1–F12.
15. Allard, S.D.; De Keersmaecker, B.; de Goede, A.L.; Verschuren, E.J.; Koetsveld, J.; Reedijk, M.L.; Wylock, C.; De Bel, A.V.; Vandeloo, J.; Pistoor, F.; et al. A phase I/IIa immunotherapy trial of HIV-1-infected patients with Tat, Rev and Nef expressing dendritic cells followed by treatment interruption. *Clin. Immunol.* **2012**, *142*, 252–268.
16. Routy, J.P.; Boulassel, M.R.; Yassine-Diab, B.; Nicolette, C.; Healey, D.; Jain, R.; Landry, C.; Yegorov, O.; Tcherepanova, I.; Monesmith, T.; et al. Immunologic activity and safety of autologous HIV RNA-electroporated dendritic cells in HIV-1 infected patients receiving antiretroviral therapy. *Clin. Immunol.* **2010**, *134*, 140–147.
17. Kongsted, P.; Borch, T.H.; Ellebaek, E.; versen, T.Z. Andersen, R.; Met, Ö.; Hansen, M.; Lindberg, H.; Sengeløv, L.; Svane, I.M.; et al. Dendritic cell vaccination in combination with docetaxel for patients with metastatic castration-resistant prostate cancer: A randomized phase II study. *Cytotherapy* **2017**, *19*, 500–513.
18. Mu, L.J.; Kyte, J.A.; Kvalheim, G.; Aamdal, S.; Dueland, S.; Hauser, M.; Hammerstad, H.; Waehre, H.; Raabe, N.; Gaudernack, G.; et al. Immunotherapy with allotumour mRNA-transfected dendritic cells in androgen-resistant prostate cancer patients. *Br. J. Cancer* **2005**, *93*, 749–756.
19. Su, Z.; Dannull, J.; Yang, B.K.; Dahm, P.; Coleman, D.; Yancey, D.; Sichi, S.; Niedzwiecki, D.; Boczkowski, D.; Gilboa, E.; et al. Telomerase mRNA-transfected dendritic cells stimulate antigen-specific CD8+ and CD4+ T cell responses in patients with metastatic prostate cancer. *J. Immunol.* **2005**, *174*, 3798–3807.
20. Heiser, A.; Coleman, D.; Dannull, J.; Yancey, D.; Maurice, M.A.; Lallas, C.D.; Dahm, P.; Niedzwiecki, D.; Gilboa, E.; Vieweg, J.; et al. Autologous dendritic cells transfected with prostate-specific antigen RNA stimulate CTL responses against metastatic prostate tumors. *J. Clin. Invest.* **2002**, *109*, 409–417.
21. Anguille, S.; Van de Velde, A.L.; Smits, E.L.; Van Tendeloo, V.F.; Juliusson, G.; Cools, N.; Nijs, G.; Stein, B.; Lion, E.; Van Driessche, A.; et al. Dendritic cell vaccination as postremission treatment to prevent or delay relapse in acute myeloid leukemia. *Blood* **2017**, *130*, 1713–1721.
22. Khoury, H.J.; Collins, R.H.Jr.; Blum, W.; Stiff, P.S.; Elias, L.; Lebkowski, J.S.; Reddy, A.; Nishimoto, K.P.; Sen, D.; Wirth, E.D. 3rd.; et al. Immune responses and long-term disease recurrence status after telomerase-based dendritic cell immunotherapy in patients with acute myeloid leukemia. *Cancer* **2017**, *123*, 3061–3072.
23. Amin, A.; Dudek, A.Z.; Logan, T.F.; Lance, R.S.; Holzbeierlein, J.M.; Knox, J.J.; Master, V.A.; Pal, S.K.; Miller, W.H.Jr.; Karsh, L.I.; et al. Survival with AGS-003, an autologous dendritic cell-based immunotherapy, in combination with sunitinib in unfavorable risk patients with advanced renal cell carcinoma (RCC): Phase 2 study results. *J. Immunother. Cancer* **2015**. *3*, 14.
24. Dannull, J.; Su, Z.; Rizzieri, D.; Yang, B.K.; Coleman, D.; Yancey, D.; Zhang, A.; Dahm, P.; Chao, N.; Gilboa, E.; et al. Enhancement of vaccine-mediated antitumor immunity in cancer patients after depletion of regulatory T cells. *J. Clin. Invest.* **2005**, *115*, 3623–3633.
25. Su, Z.; Dannull, J.; Heiser, A.; Yancey, D.; Pruitt, S.; Madden, J.; Coleman, D.; Niedzwiecki, D.; Gilboa, E.; Vieweg, J.; et al. Immunological and clinical responses in metastatic renal cancer patients vaccinated with tumor RNA-transfected dendritic cells. *Cancer Res.* **2003**, *63*, 2127–33.

26. Shindo, Y.; Hazama, S.; Maeda, Y.; Matsui, H.; Iida, M.; Suzuki, N.; Yoshimura, K.; Ueno, T.; Yoshino, S.; Sakai, K.; et al. Adoptive immunotherapy with MUC1-mRNA transfected dendritic cells and cytotoxic lymphocytes plus gemcitabine for unresectable pancreatic cancer. *J. Transl. Med.* **2014**, *12*, 175.
27. Suso, E.M.; Dueland, S.; Rasmussen, A.M.; Vetrhus, T.; Aamdal, S.; Kvalheim, G.; Gaudernack, G. hTERT mRNA dendritic cell vaccination: complete response in a pancreatic cancer patient associated with response against several hTERT epitopes. *Cancer Immunol. Immunother.* **2011**, *60,* 809–818.
28. Morse, M.A.; Nair, S.K.; Boczkowski, D.; Tyler, D.; Hurwitz, H.I.; Proia, A.; Clay, T.M.; Schlom, J.; Gilboa, E.; Lyerly, H.K. The feasibility and safety of immunotherapy with dendritic cells loaded with CEA mRNA following neoadjuvant chemoradiotherapy and resection of pancreatic cancer. *Int. J. Gastrointest. Cancer* **2002**, *32*, 1–6.
29. Reap, E.A.; Suryadevara, C.M.; Batich, K.A.; Sanchez-Perez, L.; Archer, G.E.; Schmittling, R.J.; Norberg, P.K.; Herndon, J.E.2nd.; Healy, P.; Congdon, K.L.; et al. Dendritic Cells Enhance Polyfunctionality of Adoptively Transferred T Cells That Target Cytomegalovirus in Glioblastoma. *Cancer Res.* **2018**, *78*, 256–264.
30. Batich, K.A.; Reap, E.A.; Archer, G.E.; Sanchez-Perez, L.; Nair , S.K.; Schmittling, R.J.; Norberg, P.; Xie , W.; Herndon II, J.E.; et al. Long-term Survival in Glioblastoma with Cytomegalovirus pp65-Targeted Vaccination. *Clin. Cancer Res.* **2017**, *23*, 1898–1909.
31. Mitchell, D.A.; Batich, K.A.; Gunn, M.D.; Huang, M.N.; Sanchez-Perez, L.; Nair, S.K.; Congdon, K.L.; Reap, E.A.; Archer, G.E.; Desjardins, A.; et al. Tetanus toxoid and CCL3 improve dendritic cell vaccines in mice and glioblastoma patients. *Nature* **2015**, *519*, 366–369.
32. Vik-Mo, E.O.; Nyakas, M.; Mikkelsen, B.V.; Moe, M.C.; Due-Tønnesen, P.; Suso, E.M.; Sæbøe-Larssen, S.; Sandberg, C.; Brinchmann, J.E.; Helseth, E.; et al. Therapeutic vaccination against autologous cancer stem cells with mRNA-transfected dendritic cells in patients with glioblastoma. *Cancer Immunol. Immunother.* **2013**, *62*, 1499–1509.
33. Morse, M.A.; Nair, S.K.; Mosca, P.J.; Hobeika, A.C.; Clay, T.M.; Deng, Y.; Boczkowski, D.; Proia, A.; Neidzwiecki, D.; Clavien, P.A.; et al. Immunotherapy with autologous, human dendritic cells transfected with carcinoembryonic antigen mRNA. *Cancer Invest.* **2003**, *21*, 341–349.
34. Lesterhuis, W.J.; De Vries, I.J.; Schreibelt, G.; Schuurhuis, D.H.; Aarntzen, E.H.; De Boer, A.; Scharenborg, N.M.; Van De Rakt, M.; Hesselink, E.J.; Figdor, C.G.; et al. Immunogenicity of dendritic cells pulsed with CEA peptide or transfected with CEA mRNA for vaccination of colorectal cancer patients. *Anticancer Res.* **2010**, *30*, 5091–5097.
35. Rains, N.; Cannan, R.J.; Chen, W.; Stubbs, R.S.; et al. Development of a dendritic cell (DC)-based vaccine for patients with advanced colorectal cancer. Hepatogastroenterology **2001**, *48*, 347–351.
36. Hobo, W.; Strobbe, L.; Maas, F.; Fredrix, H.; Greupink-Draaisma, A.; Esendam, B.; de Witte, T.; Preijers, F.; Levenga, H.; van Rees, B.; et al. Immunogenicity of dendritic cells pulsed with MAGE3, Survivin and B-cell maturation antigen mRNA for vaccination of multiple myeloma patients. *Cancer Immunol. Immunother.* **2013**, *62*, 1381–1392.
37. Maeda, Y.; Yoshimura, K.; Matsui, H.; Shindo, Y.; Tamesa, T.; Tokumitsu, Y.; Hashimoto, N.; Tokuhisa, Y.; Sakamoto, K.; Sakai, K.; et al. Dendritic cells transfected with heat-shock protein 70 messenger RNA for patients with hepatitis C virus-related hepatocellular carcinoma: a phase 1 dose escalation clinical trial. *Cancer Immunol. Immunother.* **2015**, *64*, 1047–1056.
38. Caruso, D.A.; Orme, L.M.; Amor, G.M.; Neale, A.M.; Radcliff, F.J.; Downie, P.; Tang, M.L.; Ashley, D.M. Results of a Phase I study utilizing monocyte-derived dendritic cells pulsed with tumor RNA in children with Stage 4 neuroblastoma. *Cancer* **2005**, *103*, 1280–1291.
39. Caruso, D.A.; Orme, L.M.; Neale, A.M.; Radcliff, F.J.; Amor, G.M.; Maixner, W.; Downie, P.; Hassall, T.E.; Tang, M.L.; Ashley, D.M. Results of a phase 1 study utilizing monocyte-derived dendritic cells pulsed with tumor RNA in children and young adults with brain cancer. *Neuro. Oncol.* **2004**, *6*, 236–246.
40. Van Craenenbroeck, A.H.; Smits, E.L.; Anguille, S.; Van de Velde, A.; Stein, B.; Braeckman, T.; Van Camp, K.; Nijs, G.; Ieven, M.; Goossens, H.; et al. Induction of cytomegalovirus-specific T cell responses in healthy volunteers and allogeneic stem cell recipients using vaccination with messenger RNA-transfected dendritic cells. *Transplantation* **2015**, *99*, 120–127.
41. Coosemans, A.; Vanderstraeten, A.; Tuyaerts, S.; Verschuere, T.; Moerman, P.; Berneman, Z.; Vergote, I.; Amant, F.; Van Gool, S.W.; et al. Immunological response after WT1 mRNA-loaded dendritic cell immunotherapy in ovarian carcinoma and carcinosarcoma. *Anticancer Res*. **2013**, *33*, 3855–3859.

42. Sioud, M.; Saeboe-Larssen, S.; Hetland, T.E.; Kaern, J.; Mobergslien, A.; Kvalheim, G. Silencing of indoleamine 2,3-dioxygenase enhances dendritic cell immunogenicity and antitumour immunity in cancer patients. *Int. J. Oncol.* **2013**, *43*, 280–288.
43. Hernando, J.J.; Park, T.W.; Fischer, H.P.; Zivanovic, O.; Braun, M.; Pölcher, M.; Grünn, U.; Leutner, C.; Pötzsch, B.; Kuhn, W. Vaccination with dendritic cells transfected with mRNA-encoded folate-receptor-alpha for relapsed metastatic ovarian cancer. *Lancet. Oncol.* **2007**, *8*, 451–454.
44. Coosemans, A.; Vanderstraeten, A.; Tuyaerts, S.; Verschuere, T.; Moerman, P.; Berneman, Z.N.; Vergote, I.; Amant, F.; VAN Gool, S.W. Wilms' Tumor Gene 1 (WT1)--loaded dendritic cell immunotherapy in patients with uterine tumors: a phase I/II clinical trial. *Anticancer Res*. **2013**, *33*, 5495–5500.
45. Van Nuffel, A.M.; Benteyn, D.; Wilgenhof, S.; Corthals, J.; Heirman, C.; Neyns, B.; Thielemans, K.; Bonehill, A. Intravenous and intradermal TriMix-dendritic cell therapy results in a broad T-cell response and durable tumor response in a chemorefractory stage IV-M1c melanoma patient. *Cancer Immunol. Immunother*. **2012**, *61*, 1033–1043.
46. Schuurhuis, D.H.; Verdijk, P.; Schreibelt, G.; Aarntzen, E.H.; Scharenborg, N.; de Boer, A.; van de Rakt, M.W.; Kerkhoff, M.; Gerritsen, M.J.; Eijckeler, F.; et al. In situ expression of tumor antigens by messenger RNA-electroporated dendritic cells in lymph nodes of melanoma patients. *Cancer Res.* **2009**, *69*, 2927–2934.
47. Kyte, J.A.; Mu, L.; Aamdal, S.; Kvalheim, G.; Dueland, S.; Hauser, M.; Gullestad, H.P.; Ryder, T.; Lislerud, K.; Hammerstad, H.; et al. Phase I/II trial of melanoma therapy with dendritic cells transfected with autologous tumor-mRNA. *Cancer Gene Ther.* **2006**, *13*, 905–918.
48. Van Tendeloo, V.F.; Van de Velde, A.; Van Driessche, A.; Cools, N.; Anguille, S.; Ladell, K.; Gostick, E.; Vermeulen, K.; Pieters, K.; Nijs, G.; et al. Induction of complete and molecular remissions in acute myeloid leukemia by Wilms' tumor 1 antigen-targeted dendritic cell vaccination. *Proc. Natl. Acad. Sci. USA* **2010**, *107*, 13824–13829.
49. Van Driessche, A.; Van de Velde, A.L.; Nijs, G.; Braeckman, T.; Stein, B.; De Vries, J.M.; Berneman, Z.N.; Van Tendeloo, V.F. Clinical-grade manufacturing of autologous mature mRNA-electroporated dendritic cells and safety testing in acute myeloid leukemia patients in a phase I dose-escalation clinical trial. *Cytotherapy* **2009**, *11*, 653–668.
50. Mitchell, D.A.; Sayour, E.J.; Reap, E.; Schmittling, R.; DeLeon, G.; Norberg, P.; Desjardins, A.; Friedman, A.H.; Friedman, H.S.; Archer, G.; et al. Severe adverse immunologic reaction in a patient with glioblastoma receiving autologous dendritic cell vaccines combined with GM-CSF and dose-intensified temozolomide. *Cancer Immunol. Res*. **2015**, *3*, 320–325.
51. Nair, S.K.; Morse, M.; Boczkowski, D.; Cumming, R.I.; Vasovic, L.; Gilboa, E.; Lyerly, H.K. Induction of tumor-specific cytotoxic T lymphocytes in cancer patients by autologous tumor RNA-transfected dendritic cells. *Ann. Surg.* **2002**, *235*, 540–549.
52. Coosemans, A.; Wolfl, M.; Berneman, Z.N.; Van Tendeloo, V.; Vergote, I.; Amant, F.; Van Gool, S.W. Immunological response after therapeutic vaccination with WT1 mRNA-loaded dendritic cells in end-stage endometrial carcinoma. *Anticancer Res.* **2010**, *30*, 3709–3714.

© 2020 by the authors. Licensee MDPI, Basel, Switzerland. This article is an open access article distributed under the terms and conditions of the Creative Commons Attribution (CC BY) license (http://creativecommons.org/licenses/by/4.0/).
